# Physical and mechanical properties of *Albizia procera* glulam beam

**DOI:** 10.1016/j.heliyon.2023.e18383

**Published:** 2023-07-17

**Authors:** Atanu Kumar Das, Md Nazrul Islam, Chayan Kumar Ghosh, Rupak Kumar Ghosh

**Affiliations:** a Department of Forest Biomaterials and Technology, Swedish University of Agricultural Sciences, SE- 90183, Umeå, Sweden; b Forestry and Wood Technology Discipline, Khulna University, Khulna, 9208, Bangladesh; c Forest Chemistry Division, Bangladesh Forest Research Institute, Sholoshahar, Chattogram, 4211, Bangladesh

**Keywords:** *Albizia procera*, Glulam, Physical properties, Mechanical properties

## Abstract

This research was done to evaluate the feasibility of using *Albizia procera* for manufacturing glulam beams. The physical and mechanical properties of the *A. procera* glulam beam were evaluated, and these properties were compared to those of the solid *A. procera* solid timber. The *A. procera* glulam beam’s physical and mechanical properties were all superior to solid *A. procera* timber. In comparison to *A. procera* solid timber, *A. procera* glulam’s density, water absorption (WA), linear expansion (LE), and thickness swelling (TS) all improved by 11.1, 48.4, 44.6, and 37.0%, respectively. Again, compared to *A. procera* solid timber, the modulus of rupture (MOR) and modulus of elasticity (MOE) of the *A. procera* glulam beam increased by 27.6 and 29.2%, respectively. Additionally, the ASTM specifications were met by the *A. procera* glulam beam. As a result, based on the properties, it is possible to make *A. procera* glulam beams as structural timber products.

## 1 Introduction

One of the earliest and most sophisticated building materials is wood [1]. Because of its improved mechanical properties and contribution to a sustainable economy, it is now more frequently used for the building of complex structural systems. However, trees' mechanical characteristics change as a result of variations in their natural growth. The use of wood rather than alternative materials presents challenges for structural engineers. For this reason, researchers have worked to create engineered wood products such as glued laminated timber (glulam), cross-laminated timber (CLT), plywood, etc., with uniform mechanical and physical properties for use as construction materials [[2], [3], [4]].

Glulam, an engineered wood product, is made from sawn or planed lumber where sawn and planed lamina are bonded with an adhesive where grain direction is parallel to each other [5,6]. For glulam, sawn lumber from fast-growing trees is used since it has a lower density [5,7]. Various methods have been used by researchers to try and enhance the characteristics of glulam [[8], [9], [10]]. Studies have been done on how the characteristics of glulam are affected by the reinforcement materials, such as steel bars, flax fiber, carbon fiber, etc. [5,7,[10], [11], [12], [13], [14], [15]], finger joint [2,16], glue line [17], number of layers [18], and assembly pattern [9,19].

However, the building industry’s growing need for renewable and ecological materials necessitates the exploration of substitute materials [[20], [21], [22], [23], [24], [25]]. Numerous hardwood [2,18,[26], [27], [28]] and softwood [3,[29], [30], [31], [32]] species have been examined by various researchers to determine whether they are suitable for the synthesis of glulams. The hardwood species *Albizia procera* is one of them. It is a species with a rapid rate of growth, and it may be found from southern South-East Asia to northern Australia and Papua New Guinea. Each year, it generates 8–12 m^3^/ha of wood. *A. procera* wood is light brown, and it has dark bands. The wood is robust and resilient by nature [33]. But up until now, there hasn’t been a solitary research on how well *A. procera* glulam beams perform. A new raw material for the bio-based building industry, may be introduced with the aid of an investigation of the *A. procera* glulam beam’s performance.

Therefore, the purpose of this study was to determine whether *A. procera* wood might be used as a raw material for the manufacture of glulam. The quality was assessed by analysing the physical and mechanical properties of the *A. procera* glulam beams. A comparison was made with the properties of *A. procera* solid timber.

## 2 Materials and methods

### 2.1 Preparation of raw material

Around 18-year-old *Albizia procera*, straight and cylindrical trees with bole heights of 24.0 m and diameters of 30 cm (DBH) were obtained from Shimakhali in Magura, Bangladesh (23°19′31.9″ N, 89°19′01.4″ E). The shortest 3.0 m long log was chosen to make the glulam and converting it into solid wood. The preparation of lumber and solid wood timbers involved cross-cutting them lengthwise with a handsaw. Lumber and solid wood timbers had a moisture level of 55–65% (dry basis). Then, to achieve a moisture content of 12 ± 2%, both types were dried using an automatic roller track timber dryer with hot air circulation for 30 min while maintaining roller rotation at 550 rpm and a temperature of 170–180 °C. Trial and error were used to choose these drying parameters in order to reach the desired moisture content level. To achieve a smooth surface, dried lumber and solid wood timbers were manually sanded with sandpaper no. 120. Solid wood timbers and dried lumber without defects were chosen for further processing. Each lumber had final dimensions of 2400 × 120 × 10 mm. Solid wood timbers with dimensions of 2400 × 120 × 100 mm were converted for comparison.

Al-Amin Plywood Industries Limited, Shimakhali, Magura, Bangladesh, provided the commercial grade urea-formaldehyde (UF) resins (48% solid content), wheat flour (extender), ammonium chloride (NH_4_Cl) (hardener), hexaconazol (preservative for protection from biodegradation), and fevicol glue (a white adhesive for the enhancement of bonding ability). The adhesive was prepared by mixing 25.0 wt% extenders, 1.1 wt% hardeners, 0.4 wt% preservative, and 0.005 wt% fevicol glue (based on the dry content of UF) into the UF resin. At first, the water (the amount of water was fixed considering the target solid content of 50%) was added to UF resin and manually stirred for 20 min. The extender was then added, and the mixture was manually stirred for an additional 7 min. Finally, the hardener and preservative were added to the mixture, and it was then stirred for 3 min. The glulam beams were prepared using the specially formulated UF adhesive with a solid concentration of 50.0 ± 0.5%.

### 2.2 Preparation of glulam beams

**Fig. 1:**
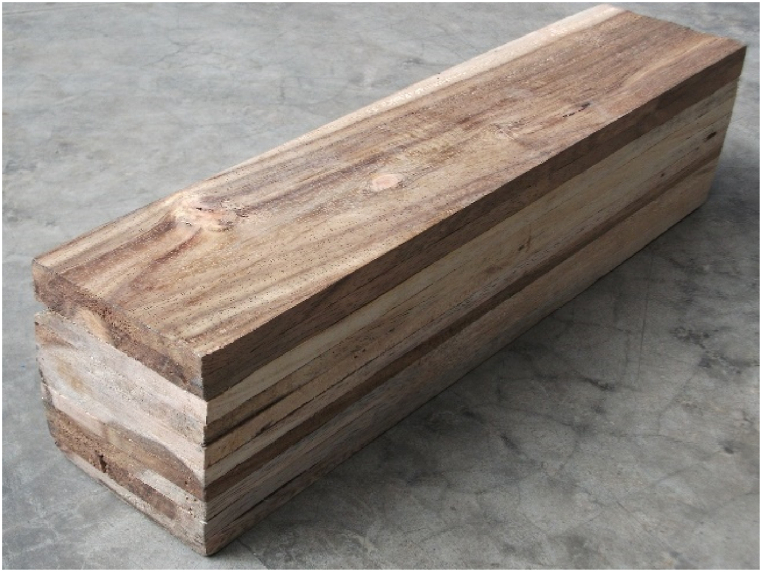
*Albizia procera* glulam beam.

Ten layers of *A. procera* lumber were used to prepare each glue-laminated timber (glulam) beam. Each glulam had longitudinally oriented lumber (Fig. 1). The above-mentioned developed UF adhesive was used to adhere these layers together. In accordance with Donadon et al. [34], gluing and pressing were performed. In this investigation, however, the pressure and pressing duration were changed. In a nutshell, lumbers were coated with adhesive at a spread rate of approximately 400 g/m^2^ per lumber. The adhesive was then allowed to settle down on the surface of the adhesive-coated lumbers by 2 h of storage at room temperature. Then, adhesive-coated lumbers were put together in the right sequence and alignment. The assembled lumber was pressed in a multi-plate hot press in two steps for 8 min, and the applied pressure was 9.0 N/mm^2^. The pressure was applied for 3 min in the first step to eliminate the air between the layers of lumber, then for 5 min at 115 °C in the following step. The *A. procera* glulam beam’s final dimension was 2400 × 120 × 100 mm. The manufactured glulam beams were conditioned in a controlled environment with a temperature of 20 ± 3 °C and 65 ± 5% relative humidity.

### 2.3 Characterization of glulam beams

The physical and mechanical properties of *A. procera* glulam beams were measured to characterize them, and both properties of *A. procera* solid timber were determined accordingly. Physical properties were investigated in the laboratory of Forestry & Wood Technology Discipline, Khulna University, Khulna, Bangladesh, while mechanical properties were tested (the test machine is shown in Fig. S1) in the laboratory of the Department of Civil Engineering, Khulna University of Engineering & Technology, Khulna, Bangladesh. The ASTM D2395-17 standard was followed while measuring the density (Eq. (1)). According to the ASTM D1037-99 standard, the linear expansion (LE) (Eq. (2)), thickness swelling (TS) (Eq. (3)), and water absorption (WA) (Eq. (4)) measurements were used to assess the dimensional stability, while the modulus of elasticity (MOE) (Eq. (5)) and modulus of rupture (MOR) (Eq. (6)) measurements were used to analyse the mechanical properties. Moisture content was also tested using the ASTM D1037-99 standard (Eq. (7)). For the mechanical (a little length modification of the standard size) and physical properties, the sample sizes were 50 × 120 × 100 mm and 300 × 120 × 100 mm, respectively. The samples were weighed before and after soaking in the water to determine LE, TS, and WA. The samples were submerged in the water for 24 h at room temperature. For this study’s analysis of both categories of physical and mechanical properties, there were ten replications.

(1) $$D=\frac{m}{b_{1}\times b_{2}\times t}$$

where, *D* = Density, *b*_*1*_ = The length of the sample (mm) and *b*_*2*_ = The width of the sample (mm) and *t* = The thickness of the sample (mm)

(2) $$LE\;\left(\%\right)=\frac{L_{A}-L_{B}}{L_{B}}\times 100$$

where, *LE* = Linear expansion, *L*_*A*_ = Length of sample after immersion (24 h) in water (mm), and *L*_*B*_ = Length of sample before immersion in water (mm)

(3) $$TS\;=\frac{t_{2}-t_{1}}{t_{1}}\times 100$$

where, *TS* = Thickness swelling (%), $t_{2}$ = Thickness of sample after immersion (24 h) in water, and $t_{1}$ = Thickness of sample before immersion in water

(4) $$WA\;=\frac{m_{2}-m_{1}}{m_{1}}\times 100$$

where, *WA*= Water absorption (%), $m_{2}$ = The weight of the sample after (24 h) immersion in water (g), and $m_{1}$ = The weight of the sample before immersion in water (g)

(5) $$MOE=\frac{P^{\prime }L^{3}}{4\Delta bd^{2}}$$

where, *MOE* = The modulus of elasticity (N/mm^2^), $P^{\prime }$ = The load in N at the limit of proportionality, *L* = The span length (mm), *Δ* = The deflection in mm at the limit of proportionality, *b* = The width of sample (mm), and *d* = The thickness/depth of sample (mm)

(6) $$MOR=\frac{3PL}{2bd^{2}}$$

where, *MOR* = The modulus of rupture (N/mm^2^), *P* = Load (N), *L* = Span length (mm), *b* = Width of test sample (mm), and *d* = Thickness of test sample (mm)

(7) $$MC\;=\frac{m_{\mathrm{int}}-m_{od}}{m_{od}}\times 100$$

where, *MC* = Moisture content (%), $m_{\mathrm{int}}$ = Initial mass of the sample (g), and $m_{od}$ = Oven-dry mass of the sample (g).

### 2.4 Analysis

SPSS (Statistical Package of Social Survey) software was used to evaluate all the data generated during the laboratory tests for the evaluation of the physical and mechanical properties of *A. procera* glulam beam and *A. procera* solid timber. A spreadsheet was used to build a graphical presentation.

## 3 Results and discussion

### 3.1 Physical properties

**Fig. 2:**
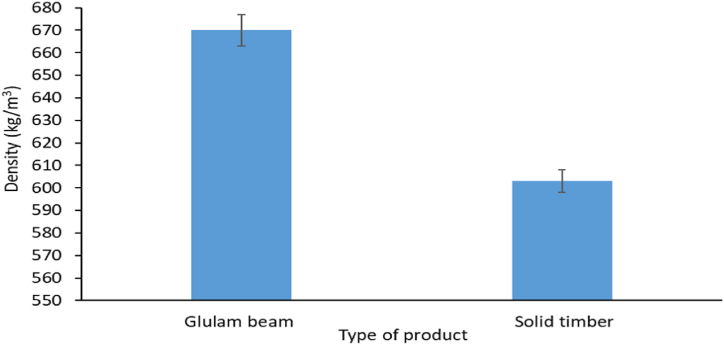
Density of *Albizia procera* glulam beam and solid timber.

The measured densities of the *Albizia procera* glulam beam and *A. procera* solid timber were 670 and 603 kg/m^3^, respectively (Fig. 2). The density of the *A. procera* glulam beam was found to be 11.1% higher than that of the *A. procera* solid timber. The density and confinement of the fibres are both increased by the applied pressure [20,35,36]. Additionally, the density is affected by the adhesive. As a result, the density of the *A. procera* glulam beam increased. Pressure and temperature may also aid in increasing the densification of glulam. The ASTM criteria (430–794 kg/m^3^) were met by the density of the *A. procera* glulam beam [37]. It was also substantially higher than glulam beams made of Douglas-Fir-Larch (500 kg/m^3^), southern pine (550 kg/m^3^), and Spruce-Pine-Fir (420 kg/m^3^) [38]. According to the statistical analysis, the density of the *A. procera* glulam beam differed significantly (p < 0.05) from that of the solid *A. procera* timber.

**Fig. 3:**
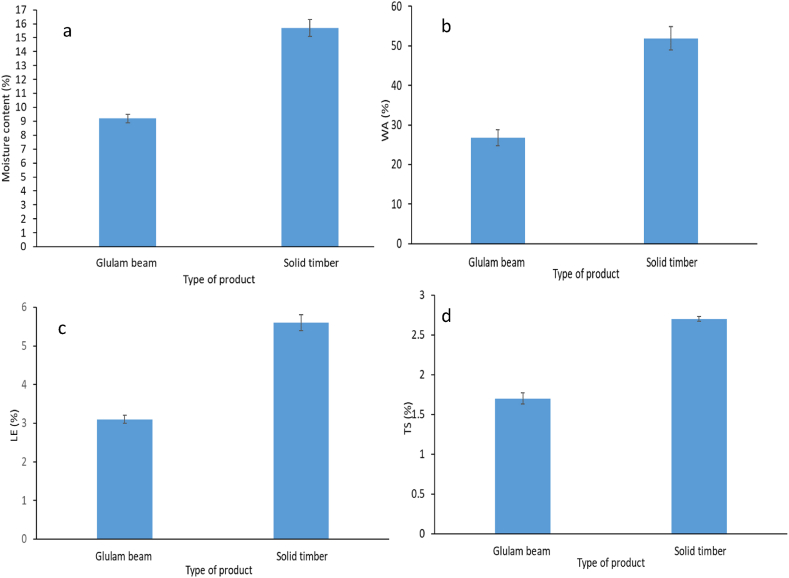
(a) Moisture content, (b) Water absorption (WA), (c) Linear Expansion (LE), and (d) Thickness swelling (TS) of *Albizia procera* glulam beam and solid timber.

The measured value for the moisture content of *A. procera* glulam beam and *A. procera* solid timber is shown in Fig. 3a. Compared to solid *A. procera* solid timber, which had a moisture content of 15.7%, the *A. procera* glulam beam had a moisture content of 9.2%. The moisture content of the *A. procera* glulam beam was decreased by the glulam manufacturing process and was significantly (p < 0.05) lower than the moisture content of the *A. procera* solid timber. In the current work, the applied temperature during assembly may have had a significant impact on lowering the moisture content of the *A. procera* glulam beam. It fell within the range (8.3–11.9%) that other studies had noted for *Guadua angustifolia* Kunth glulam beams [20]. Additionally, it adhered to the moisture content range (7.3–12.7%) of the ASTM specifications [37].

As observed in Fig. 3b, after soaking in water for 24 h, the water absorption (WA) of the *A. procera* glulam beam (26.8%) was lower than that of the *A. procera* solid timber (51.9%). According to statistical analysis, the WA for *A. procera* glulam beam was significantly (p < 0.05) lower than that for *A. procera* solid timber. The *A. procera* glulam beam’s WA property was 48.4% better thanks to the glulam technology. Applying pressure encourages glulam’s cross-linking and induces cross-linked polymerization, which lowers WA [35]. The ability of water repellent of wood is improved by the penetration of adhesive into the wood’s pores, which may close off the space where water molecules could otherwise enter.

The linear expansion (LE) of the *A. procera* glulam beams and *A. procera* solid timber was 3.1 and 5.6%, respectively, after soaking in water for 24 h (Fig. 3c). The LE for the *A. procera* glulam beams was significantly (p < 0.05) lower. The LE for the *A. procera* glulam beams was decreased by 44.6%. The LE of the *A. procera* glulam beam may drop due to the reason for preventing water absorption that was discussed previously.

The thickness swelling (TS) of the *A. procera* glulam beams and *A. procera* solid timber is shown in Fig. 3d. *A. procera* glulam beams and *A. procera* solid timber had measured TS values of 1.7 and 2.7%, respectively. The TS of the *A. procera* solid timber vs *A. procera* glulam beams differed significantly (p < 0.05). In comparison to solid *A. procera* timber, the TS reduction of the *A. procera* glulam beam was 37.0%. Applying pressure causes the adhesive and wood in glulam to cross-link polymerize, which also lowers the glulam’s TS [35]. In addition, the use of adhesive acts as a TS restriction for engineered products [39]. Therefore, the rigid structure formed by pressing after adhering may lower the TS value of the *A. procera* glulam beams.

### 3.2 Mechanical properties

**Fig. 4:**
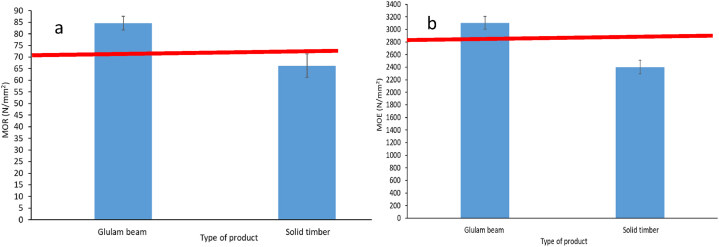
(a) Modulus of Rupture (MOR) and (b) Modulus of Elasticity (MOE) of *Albizia procera* glulam beam and solid timber. Red line indicates the minimum value according the standard. (For interpretation of the references to colour in this figure legend, the reader is referred to the Web version of this article.)

Fig. 4a shows the measured modulus of rupture (MOR) values for the *A. procera* glulam beam and *A. procera* solid timber. Compared to *A. procera* solid timber (66.3 N/mm^2^), MOR of the glulam beam (84.6 N/mm2) was significantly greater (p < 0.05). Compared to *A. procera* solid timber, MOR of the *A. procera* glulam beam rose by 27.6%. The increase in fibre density and confinement of fibres are influenced by lamination and pressure [20,35,40]. A greater value of MOR is also caused by the layered structure and lumber that is arranged parallel to the grain in glulam [4]. Once again, pressure that is provided can improve the MOR of glulam [3,35,36]. Mechanical properties are correlated with density. A thicker cell wall produces a larger density by enhancing the compressive strength along the fibers [26]. *A. procera* glulam beams had a higher density than *A. procera* solid wood timber. A thick cell of *A. procera* wood may form as a result of the pressure that is applied to manufacture glulam. As a result, the MOR value of the *A. procera* glulam beams was greater. The MOR of white ash and birch glulam was previously reported to be 39.7–47.0 N/mm^2^ [2] and 19.9–28.2 N/mm2 for *Toona ciliate* wood [18]. The *A. procera* glulam beam had higher MOR in this investigation than in other studies. The ASTM requirements (72.0–100.0 N/mm^2^) were followed for the MOR of *A. procera* glulam beams [37].

*A. procera* glulam beams and *A. procera* solid timber were found to have modulus of elasticity (MOE) of 3104.3 and 2402.5 N/mm^2^, respectively (Fig. 4b). The MOE of the *A. procera* glulam beam was greater than that of the *A. procera* solid timber, and the differences between the MOE values were significant (p < 0.05). Comparing the *A. procera* glulam beam to the *A. procera* solid timber, the MOE improvement was 29.2%. The MOE can be improved by increasing the density and confinement of fibres in the glulam as a result of pressure during the lamination process [20]. Once more, glulam’s lay-up structure and parallel-to-grain lumber arrangement improve MOE [4]. Additionally, applying pressure [3,35,36] and adhesive results in an improvement of the glulam’s compactness. Furthermore, mechanical properties are proportional to density [26], and *A. procera* glulam beam displayed a greater density value. As a result, higher MOE value was achieved for *A. procera* glulam beam. In the future, investigations using scanning electronic microscopy (SEM) and Fourier-transform infrared spectroscopy (FTIR) will be able to better explain how glue penetrates wood of *A. procera* glulam beam and interacts with it. The MOE of white ash and birch glulam was measured at 15157.3–16593.9 N/mm^2^ [2] in earlier studies, and it ranged from 548.0 to 2798.7 N/mm^2^ for *T. ciliate* wood [18]. In contrast to other research, the *A. procera* glulam beam in this investigation had a lower and higher MOE value. The MOE of the *A. procera* glulam beam followed the ASTM standard (2890–4100 N/mm^2^) [37] as well.

## 4 Conclusions

This study examined the properties of a glulam beam obtained from the *Albizia procera* lumber. The physical and mechanical properties of *A. procera* glulam beam and *A. procera* solid timber were compared. When compared to *A. procera* solid timber, density and mechanical properties of *A. procera* glulam beam were much greater. Moisture content, water absorption, linear expansion and thickness swelling of *A. procera* glulam beam were much lower compared to *A. procera* solid timber. In addition, the *A. procera* glulam beam also fulfilled ASTM specifications. Further research is required to determine how the number of lumber used, the drying effect of the lumber, and the reinforcement of the material affect the properties of the *A. procera* glulam beam.

## Funding

None.

## Author contribution statement

Atanu Kumar Das: Conceived and designed the experiments; Performed the experiments; Analyzed and interpreted the data; Wrote the paper. Md. Nazrul Islam: Conceived and designed the experiments; Contributed reagents, materials, analysis tools or data; Wrote the paper. Chayan Kumar Ghosh: Performed the experiments; Analyzed and interpreted the data; Wrote the paper. Rupak Kumar Ghosh: Analyzed and interpreted the data; Wrote the paper.

## Data availability statement

The data that has been used is confidential.

## Declaration of competing interest

The authors declare that they have no known competing financial interests or personal relationships that could have appeared to influence the work reported in this paper
