# Supplementary figures and images for: Physical and mechanical properties of Albizia procera glulam beam
Source: Heliyon. 2023 Jul 17;9(8):e18383. doi: 10.1016/j.heliyon.2023.e18383 (PMC10382284; doi:10.1016/j.heliyon.2023.e18383)

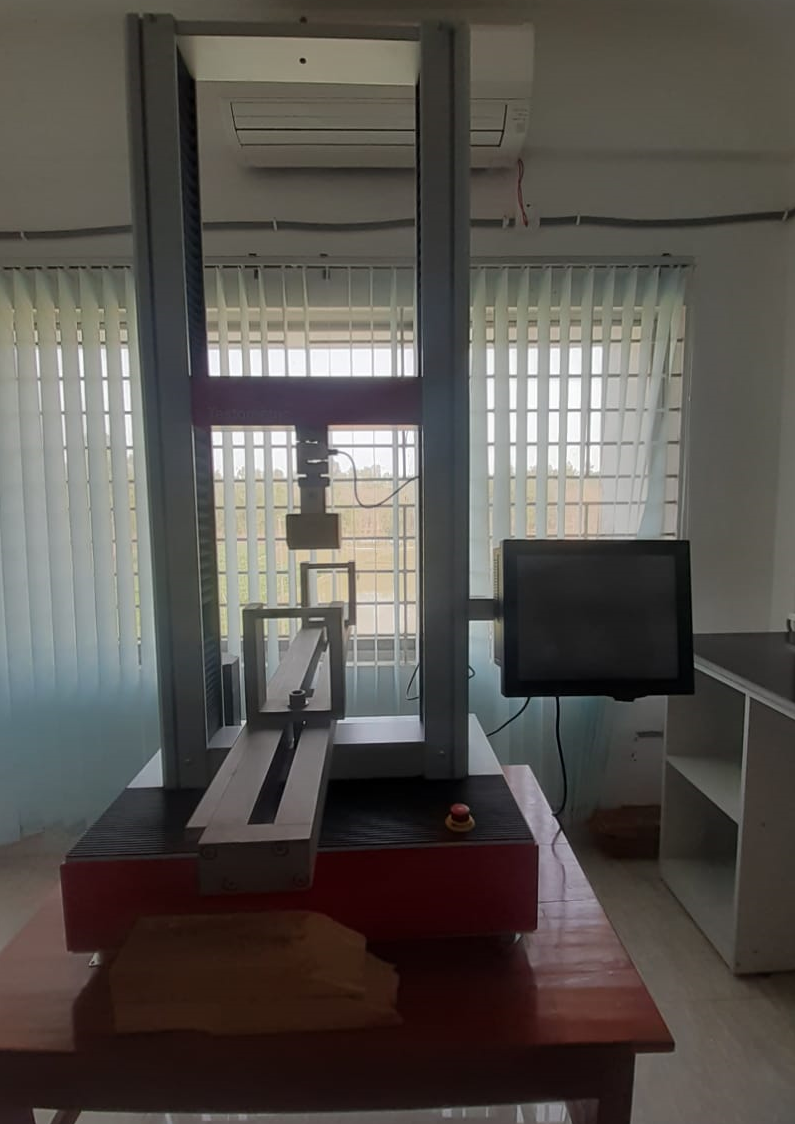


Fig. S1: Mechanical properties test machine.

Supplement: Multimedia component 1 [file mmc1.docx]
